# Cancer stem cells and signaling pathways in radioresistance

**DOI:** 10.18632/oncotarget.6760

**Published:** 2015-12-24

**Authors:** Lei Chang, Peter Graham, Jingli Hao, Jie Ni, Junli Deng, Joseph Bucci, David Malouf, David Gillatt, Yong Li

**Affiliations:** ^1^ Cancer Care Centre, St George Hospital, Kogarah, NSW, Australia; ^2^ St George and Sutherland Clinical School, Faculty of Medicine, University of New South Wales, Kensington, NSW, Australia; ^3^ Department of Urology, St George Hospital, Kogarah, NSW, Australia; ^4^ Australian School of Advanced Medicine, Macquarie University, Sydney, NSW, Australia

**Keywords:** cancer, radiotherapy, radioresistance, CSC, signaling pathway

## Abstract

Radiation therapy (RT) is one of the most important strategies in cancer treatment. Radioresistance (the failure to RT) results in locoregional recurrence and metastasis. Therefore, it is critically important to investigate the mechanisms leading to cancer radioresistance to overcome this problem and increase patients' survival. Currently, the majority of the radioresistance-associated researches have focused on preclinical studies. Although the exact mechanisms of cancer radioresistance have not been fully uncovered, accumulating evidence supports that cancer stem cells (CSCs) and different signaling pathways play important roles in regulating radiation response and radioresistance. Therefore, targeting CSCs or signaling pathway proteins may hold promise for developing novel combination modalities and overcoming radioresistance. The present review focuses on the key evidence of CSC markers and several important signaling pathways in cancer radioresistance and explores innovative approaches for future radiation treatment.

## INTRODUCTION

**Figure 1:**
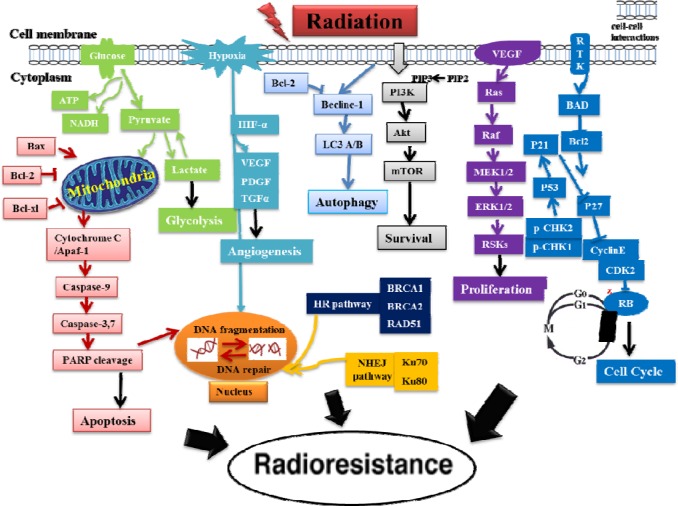
A schematic diagram for the putative mechanisms of the acquired cancer radioresistance after RT This diagram shows that the induced cancer radioresistance is associated with the activation of several pathways (PI3K/Akt/mTOR, ERK, glycolysis, VEGF, autophagy, NHEJ and HR DNA repairs), the induction of cell cycle redistribution and inactivation of apoptosis pathway after exposure to radiation. Notes: ERK: Extracellular signal-regulated kinases, HR: Homologous recombination, NHEJ: Non-homologous end joining, VEGF: Vascular endothelial growth factor.

Radiation therapy (RT) is one of the most important strategies to kill cancer cells and shrink tumor. Approximately 50% of all patients with cancer receive RT at some points in their treatment, alone or in combination with surgery and/or chemotherapy [1]. However, radioresistance and cancer recurrence are major obstacles for the long-term survival of patients undergoing RT [2, 3]. Thus, understanding the mechanisms of radioresistance is important for the improvement of RT. The mechanisms of cancer radioresistance are very complicated and affected by many factors, which severely affect radiation efficacy. One possible reason for RT failure may be the intrinsic radioresistance of a subpopulation of clonogenic cells within the tumor [4] while another reason could be the acquired radioresistance during RT [5, 6]. The possible mechanisms of the acquired cancer radioresiatance are depicted in Figure 1.

Recently, cancer stem cell (CSC) theory has offered a potential explanation for the relapse and resistance that occur in many tumors [7]. Several lines of evidence support CSCs are associated with radiosensitivity [8-10]. In addition, different signaling pathways were reported to play important roles in cancer radioresistance [8, 11].

The roles of cellular hypoxia, tumor niches, tumor microenvironment, apoptosis and autophagy in cancer radioresistance have been recently reviewed [2, 12]. Here, we focus on discussing the roles of CSCs and signaling pathways in cancer radioresistance and explore novel therapeutic modalities for future cancer RT.

## CSCS IN RADIORESISTANCE

### Roles of CSCs in RT

Despite continuous improvements in cancer management, locoregional recurrence or metastatic spread still occurs in a high proportion of patients after RT or combined treatments [13]. One underlying reason might be a low efficacy of current treatments on the eradication of CSCs. Despite the ongoing debate on the abundance and origin of CSCs, it is generally accepted that they represent the root of cancer that must be eradicated in order to cure cancer. Increasing evidence indicates that CSCs contribute to radioresistance which could result in radiation treatment failure [14]. Further studying CSCs features found that CSCs led to radioresistance, which is associated with intrinsic determinants (DNA repair capability, reactive oxygen species (ROS) levels, cell cycle status, autophagy, apoptosis, regulation of survival pathway) and extrinsic determinants (the influence of hypoxic microenvironment) [14, 15]. Thus, investigation of CSCs has been a hot spot of basic cancer research and is rapidly expanding into many related aspects of cancer research, including radiosensitization [8].

### Cell cycle, DNA repair and ROS mechanisms are associated with CSCs in radioresistance

Generally, CSC subpopulations are more related to radioresistance compared to non-CSC subpopulations [15]. One representative study demonstrated that CSC marker CD133 represented a relatively radioresistant (RR) cellular population in glioblastoma (GBM) and these CSCs were particularly resistant to radiation [16]. The role of cell cycle in radioresistance is important. The activation of cell cycle checkpoint kinases, Chk1 and Chk2, was found in CD133^+^ CSCs compared with CD133^−^ non-CSCs [17]. It was reported that U87 and U251 glioma stem cells were more RR compared to glioma cells due to high expression of phosphorylated cell cycle checkpoint proteins [18]. Activation of Chk1/2 checkpoint proteins were also found in PC-3RR, DU145RR and LNCaPRR CaP cell lines [19, 20]. Inhibition of cell cycle check point protein Chk1 was found to increase radiosensitivity in CD133^+^CD44^+^ DU145 CaP cells [21]. In laryngeal carcinoma, more cells were arrested in the G_0_/G_1_ phase of the cell cycle in CD133^+^ Hep-2 cells with radiation treatment, compared to no treatment control [22]. CD133^+^ Huh-7 liver CSCs also showed greater distribution in G_0_/G_1_ phase than that of CD133^−^ cells post RT [23]. Hence, abnormal expression of cell cycle related proteins may promote CSCs proliferation after radiation. These results support that cell cycle plays an important role in CSC-associated radioresistance.

DNA repair is also involved in CSC-associated radioresoistance. It was reported that enriched CSC cell populations such as CD44^+^/CD24^−^ breast cancer cells and CD133^+^ glioma cells showed increasing DNA repair capability compared with non-CSC-enriched cell populations [24, 25]. Recent evidence also demonstrated that glioma CSCs play a crucial role in radioresistance through activation of DNA damage checkpoint proteins including ATM, SMC1, Chk1, Chk2, and p53 and increased DNA repair [26]. Zhang et al showed that CSCs exhibited more efficient DNA damage repair than bulk tumor cells when exposed to radiation [27]. Gene microarray demonstrated an increased DNA damage response and expression of DNA repair genes among Lin^(−)^CD29^(H)^CD24^(H)^ breast cancer CSC [28]. The findings from our group also suggest that more efficient DNA repair capacity in the CD44^high^ prostate cancer (CaP) cells after radiation contributes to their survival [29].

ROS plays crucial roles in cell proliferation, differentiation, metabolism, cell death, and tumorigenesis [30-32]. ROS is one of the most important regulatory mechanisms for CSCs and CSCs overexpress ROS scavengers, in order to protect them from ROS-induced damage [33-35]. The lower levels of ROS in subsets of CSCs in some tumors compared to non-CSCs populations, potentially result in increased levels of free-radical scavenging substances, which may contribute to tumor radioresistance [35]. Pharmacological depletion of ROS scavengers in Thy1^+^ CD24^+^ Lin^−^ CSC-enriched breast cells markedly decreases their clonogenicity and results in radiosensitization [35]. Blazek et al reported that CD133^+^ Daoy medulloblastoma cells were more RR than CD133^−^ cells and enlargement of the CD133^+^ subpopulation by hypoxia resulted in a low level of ROS in CSCs [36], indicating ROS level is associated with CSC and radioresistance. Kim *et al* showed that higher expression of DNA repair and greater number of low-to-intermediate ROS cells after radiation were found in LNCaP prostate spheres (CSCs) compared with adherent LNCaP cells (non-CSCs), further confirming the importance of DNA repair mechanism and ROS level in CaP [37].

All these reports support that cell cycle, DNA repair capability and ROS contribute to CSC-associated radioresistance.

### Apoptosis and autophagy are linked with CSCs in radioresistance

Apoptosis is an indispensable factor in CSCs after radiation. We recently demonstrated reduced apoptosis in CaP RR cells and enhanced CSC phenotypes at the same time [8]. Lee *et al* reported that 14-3-3ζ knockdown with short hairpin RNA (shRNA) enhanced radio-induced apoptosis by reducing radioresistance in CD133^+^ Huh7 liver cancer cell lines [38]. CD133^+^ Huh-7 liver CSCs were found to have greater anti-apoptotic activity through increased Bcl-2 expression and radioresistance [23]. The CD133^+^ thyroid cancer cells also showed higher anti-apoptotic rate after radiation [39]. Dahan *et al* demonstrated that radiation induced reprogramming in glioblastomas stem-like cells from patients was associated with the up-regulation of the anti-apoptotic protein survivin [9]. In breast cancer, the increased radioresistance in HER2^+^/CD44^+^/CD24^−/low^ MCF7 cells was found to be correlated with significantly reduced apoptosis [40].

In recent years, the role of autophagy as an alternative cell death mechanism has been a topic of debate. Autophagy was believed as a non-apoptotic programme of cell death or “type-II” cell death to distinguish from apoptosis [41]. In cancer therapy, the role of autophagy is paradoxical, in which this cellular process may serve as a pro-survival or pro-death mechanism to counteract or mediate the cytotoxic effect of anticancer agents [42]. To date, there is only little evidence for the role of autophagy in CSC-associated radioresistance. It was found that radiosensitivity of glioma stem cells can be increased by inhibiting autophagy-related proteins Becline-1 and ATG5, indicating that the induction of autophagy contributes to radioresistance of glioma stem cells [43]. Our recent data support that CaP radioresistance is associated with apoptosis and autophagy pathways and that autophagy promotes CaP RR cell survival [20].

**Figure 2:**
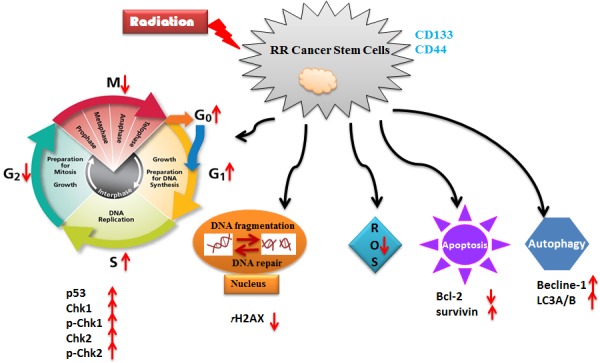
A schematic diagram for the mechanisms of CSCs in radioresistance This diagram shows the possible roles of cell cycle (p53, p-Chk1, p-Chk2), DNA repair protein (γH2AX), ROS, apoptosis (Bcl-2 and survivin) and autophagy (Becline-1 and LC3A/B) in CSC-associated radioresistance. Notes: RR: radioresistant; ROS: reactive oxygen species.

All above-mentioned findings imply that multiple mechanisms contribute to CSCs in radioresistance and targeting CSC markers or these mechanisms holds promise to overcome cancer radioresistance and improve radiosensitivity. The possible roles of cell cycle, DNA repair, ROS, apoptosis and autophagy in CSC-associated radioresistance is shown in Figure 2. The putative CSC makers in radioresistance are summarized in Table 1. All researches provide a vision that CSCs regulate radioresistance.

**Table 1:** CSC markers in cancer radioresistance

CSC marker	Source	Cancer type	Radiation dose	Testing approaches	References
CD133	CD133^+^NSC11 and GBMJ1 cells, CD133^+^NSC11 and GBMJ1 xenografts	Glioblastoma	2.9 Gy	Immunofluorescence	[16]
CD24^−/low^/CD44^+^	MCF-7 and MDA-MB-231 cells	Breast cancer	3 Gy for 5 consecutive days	Flow cytometry	[24]
CD133	CD133^+^glioma cells	Glioma cancer	5Gy	Western blot, IHC	[44]
CD29, CD24	Lin^−^CD29^+^CD24^+^ cells from p53 null mouse mammary tumors	Breast cancer	6 Gy for 2 days	IHC, qPCR, Western blot	[27, 28]
CD44	PC-3, PC-3M-luc, and LNCaP cells	Prostate cancer	0-8Gy	Flow cytometry, Western blot, immunofluorescence	[29]
CD44, CD24, CD45, CD3, CD20, CD10, glycophorin A, CD3, CD64	CD44^+^CD24^−/low^Lin-breast CSC, Thy1^+^CD24^+^Lin-cell line and xenografts	Breast cancer, head and neck cancer	10Gy (cells), 3 × 5Gy or 5 × 2Gy (xenografts)	qRT–PCR, flow cytometry	[35]
CD133	Daoy and D283 Med cell lines	Medulloblastoma	0-10Gy	Flow cytometry, survival assay	[36]
CD133	LNCaP cell line	Prostate cancer	10Gy	Western blot, flow cytometry, IHC	[37]
CD133	U87 and U251 cell lines	Glioma cancer	2-8Gy	Western blot, flow cytometry, immunofluorescence	[18]
CD133	Cells from GBM patients	Glioma cancer	3Gy	Western blot	[17]
CD133, CD44	DU145 cell line	Prostate cancer	8Gy	Western blot, RT-PCR, flow cytometry	[21]
CD133	Hep-2 cell line	laryngeal carcinoma	10Gy	Flow cytometry, immunofluorescence	[22]
CD133	Huh-7 cell line	hepatocellular carcinoma	0-20Gy	Western blot, flow cytometry	[23]
CD44, CD44v6, CD326, ALDH1	PC-3, DU145 and LNCaP cell lines	Prostate cancer	6Gy	Western blot, qRT–PCR, immunofluorescence	[20]
CD133	CD133+ cell line from GBM specimens	Glioma cancer	5Gy	Western blot, WST-1 assay, neurosphere formation assay, immunoblot analysis	[43]
CD133	Huh7 CD133+ cell line	Liver cancer	15Gy	Immunofluorescence, Western blot	[38]
CD133	WRO, CGTH, CG3, ARO cell lines	Thyroid cancer	10 or 20Gy	Western blot, flow cytometry, RT–PCR	[39]
CD44	MCF7 cell line	Breast cancer	2Gy for 5 times (xenograft)	Western blot, IHC	[40]

Notes: IHC, immunohistochemistry; qRT-PCR, Quantitative real time-PCR; RT-PCR, reverse transcription polymerase chain reaction

## SIGNALING PATHWAYS IN CANCER RADIORESISTANCE

**Figure 3:**
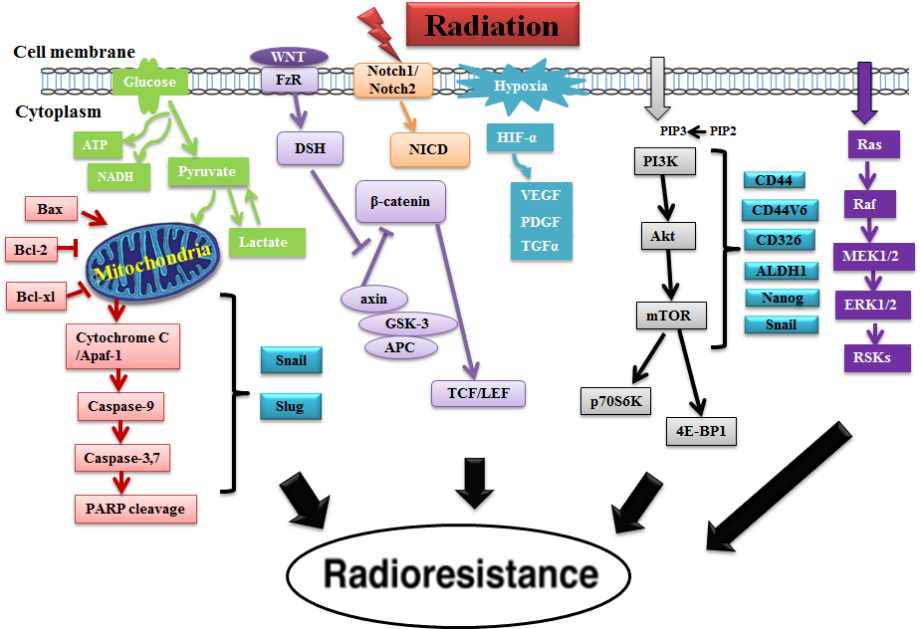
The roles of different signaling pathways associated with CSCs in radioresistance This diagram shows that cancer radioresistance is associated with several different pathways (PI3K/Akt/mTOR, ERK, glycolysis, VEGF, Notch and WNT/β-catenin pathway) as well as CSCs. We have recently demonstrated that the PI3K/Akt/mTOR signaling pathway is associated with the regulation of CSC phenotypes (CD44, CD44v6, CD326, ALDH1, Nanog and Snail) in CaP radioresistance [8]. Notes: FzR: Frizzled receptor.

Accumulating evidence from human cancer tissues and preclinical studies indicates that different signaling pathways play a critical role in cancer progression, metastasis and chemo/radioresistance via the activation of the pathway proteins or mutation, deletion, epigenetically silence of some pathway genes [8, 45]. Understanding the signaling pathways that determine radioresistance is vital for selecting appropriate treatment modalities for patients and developing novel molecular agents to enhance radiosensitivity in human cancers. In this section, we focus on several important signaling pathways that are highly associated with cancer radioresistance and also discuss the link of CSCs with these signaling pathways in radioresistance. The roles of different signaling pathways associated with CSCs in radioresistance are shown in Figure 3.

### PI3K/Akt/mTOR pathway

PI3K/Akt/mTOR pathway plays an important role in cell growth and proliferation, and is often dysregulated in cancer due to mutation, amplification, deletion, methylation and post-translational modifications. This pathway is an intracellular signaling pathway important for apoptosis, malignant transformation, tumor progression, metastasis and radioresistance [8, 46]. Datta *et al* found that radiation could persistently activate mTOR via PI3K/Akt pathway in mouse intestine [47]. Skvortsova *et al* reported that radioresistance in CaP is accompanied by the activation of the PI3K/Akt/mTOR pathway [11]. Similarly, our recent study also found the PI3K/Akt/mTOR signaling pathway is associated with CaP radioresistance in CaP RR cell lines and enhanced CSC phenotypes (CD44, CD44v6, CD326, ALDH1, Nanog and Snail) [8] (Figure 3). We further confirmed that enhanced CSCs is regulated by the PI3K/Akt/mTOR pathway [8]. Zhu *et al* demonstrated that a dual PI3K/mTOR inhibitor BEZ235 prominently improved the radiosensitivity of PC-3 CaP cells [48]. Our recent results showed that combination of dual PI3K/mTOR inhibitors (BEZ235 or PI103) with RT could overcome CaP radioresistance *in vitro* [20]. We also found that knockdown of CSC marker EpCAM with small interfering RNA (siRNA) could down-regulate the PI3K/Akt/mTOR pathway proteins and enhance radiosensitivity in CaP cells *in vitro* [49]. It was found that radiation activated the Akt/mTOR/4EBP/eIF4E signaling pathway in the A549 lung cancer cell line [50]. Heavey *et al* recently reviewed that inhibition of this pathway in non-small cell lung cancer (NSCLC) might result in the improvement of RT and overcome radioresistance [51]. Kim *et al* reported that radiation bestowed activation of the PI3K/Akt/mTOR pathway upon lung cancer by inducing hypoxia-inducible factor-1α (HIF-1α) and blocking HIF-1α could circumvent radioresistance in lung cancer cells [52].

The PI3K/Akt/mTOR pathway was reported to play important roles in radiation-induced autophagy in glioma cells [53]. Liu *et al* demonstrated that two dual PI3K/mTOR inhibitors, GSK2126458 and PKI-587, suppressed tumor progression and increased radiosensitivity in nasopharyngeal carcinoma (NPC) [54]. Chen *et al* found that the dual PI3K/mTOR inhibitor BEZ235 with radiation enhanced the radiosensitivity of colorectal cancer cells both *in vitro* and *in vivo* [55]. Mehta *et al* recently showed that using a low dose of AKT-i (Akt inhibitor) could sensitize radiation in primary human glioblastoma stem-like cells *in vitro* [56]. Therefore, the PI3K/Akt/mTOR pathway is an important pathway in the regulation of cancer radioresponse.

### ERK pathway

**Figure 4:**
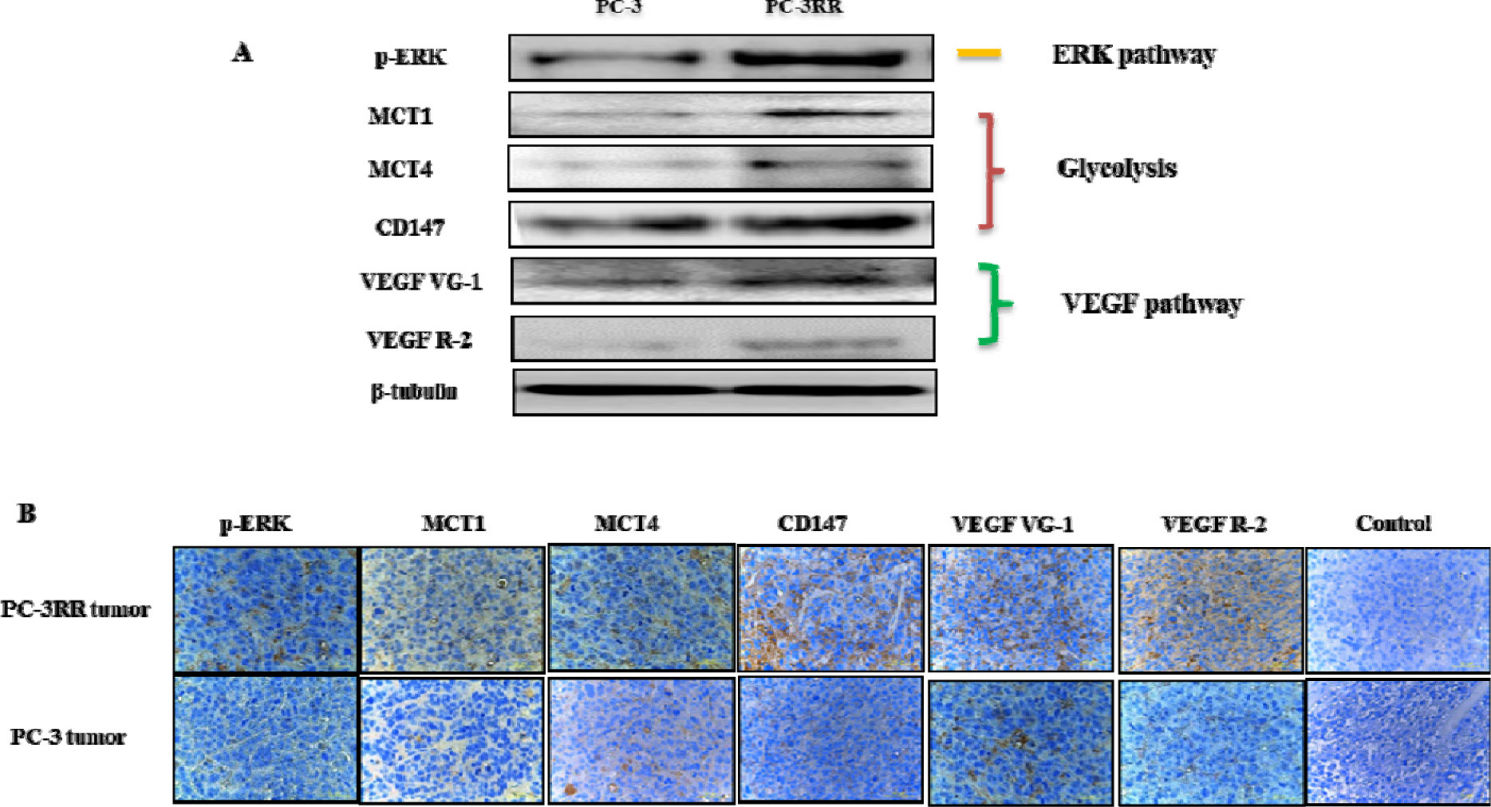
Overexpression of key proteins from ERK, Glycolysis, VEGF pathways observed in PC-3RR cell line and PC-3RR s.c xenograft tumors **A**. The representative images showing increased expression of p-ERK, MCT1, MCT4, CD147, VEGF VG-1 and VEGF R-2 (pathway associated proteins) in PC-3RR CaP cells compared with PC-3 cells by Western blotting. *β*-tubulin was used as a loading control. **B**. Representative images showing increased expression of p-ERK, MCT1, MCT4, CD147, VEGF VG-1 and VEGF R-2 in PC-3RR s.c. xenografts compared to parental PC-3 xenografts using immunohistochemistry. Brown indicates positive staining while blue indicates nuclear staining. Magnification in all images x 40. All data were from three independent experiments (n=3).

Extracellular signal-regulated kinases (ERK) pathway (also known as the mitogen-activated protein kinases (MAPK)/ERK pathway or Ras-Raf-MEK-ERK pathway) is a chain of proteins that communicates from the cell surface to the DNA and regulates the development of many cancers. ERK pathway has become a major player in response to DNA damage. Increasing evidence has shown that this pathway is related to the cellular response to ionizing radiation (IR), suggesting a role in radioresistance (Figure 3) [57]. ERK pathway was found to be involved in HER2-overexpressing breast cancer cell radioresistance [58]. Inhibition of ERK was correlated with the reduced cell growth and clonogenic survival in human RR esophageal carcinoma cells KYSE-150RR [59]. Xie *et al* reported that radiosensitivity was regulated by inactivation of ERK pathway proteins including p38, ERK, JNK in A375 human melanoma cells [60]. Marampon *et al* showed that ERK pathway, through the sustained expression of DNA-PKcs, positively regulated HIF-1α protein expression and activity, preserving GBM radioresistance in hypoxic condition [61]. Huang *et al* found that the phosphorylation of Raf-1 and ERK was up-regulated in RR cervical cancer cell lines HeLa and C33A [62]. The MEK/ERK cascade can also regulate tumor radioresistance in a panel of gynecological cancer cell lines including Ishikawa (endometrial cancer), HeLa (cervical cancer), CASKI (cervical cancer) and SiHa (cervical cancer) cell lines [63]. It was reported that this pathway was activated in head and neck squamous cell carcinoma (HNSCC) radioresistance and the expression of p-ERK was found to be decreased by MAPK inhibitor U0126 combined with radiation treatment with increased radiation response [64]. Affolter *et al* also showed that U0126 significantly reduced the level of p-ERK and suppressed colony forming ability in radiated oral squamous cancer cells HNSCCUM-02T and lung cancer cells A549 [65]. However, Gupta *et al* claimed that inhibition of the Raf-MEK-MAPK pathway with PD98059, or the Ras-MEK kinase-p38 pathway with SB203580 had no effect on radiation survival in cells with oncogenic Ras gene [66]. The reason could be that although the Raf-MAPK pathway might be a mediator of altered radiosensitivity in some systems, it was not the downstream mediator of Ras-induced radioresistance [66]. Unlike this study, our data indicate ERK pathway (overexpression of p-ERK) is activated in PC-3RR cells and PC-3RR subcutaneous (s.c) xenograft tumors (Figure 4), respectively, indicating that ERK pathway activation does contribute to radioresistance in CaP. These studies support that targeting ERK pathway may enhance the efficiency of RT.

### Glycolysis pathway

Glycolysis pathway, located in the cytoplasm of eukaryotic cells, is the metabolic pathway that is responsible for the production of adenosine triphosphate (ATP) through the degradation of glucose. Tumor malignancy could be caused by energy metabolism resulting from the increased glycolytic pathway [67]. Recent reports demonstrate that CSCs rely on glycolysis pathway in several cancers including breast cancer, laryngeal cancer, nasopharyngeal cancer as well as glioma [68-71]. In our study, glycolysis pathway (overexpression of MCT1, MCT4 and CD147) was found to be activated in PC-3RR cell line and PC-3RR CaP xenograft tumor tissues (Figure 4). Bing *et al* reported that glycolysis might impede radiation treatment in RR HeLa cervical cancer cell line [72]. It was found that expression of glucose transporter Glut1 and lactate production rate were increased in RR HepG2 liver cancer cells and HeLa cervical cancer cells compared with parental cells, respectively [73]. One study indicated that RR OE33 esophageal adenocarcinoma cells showed significantly increased intracellular ATP levels and the expression of ATP5B, HSP60, GAPDH as well as pyruvate kinase M2 isoform (PKM2) proteins, suggesting the activation of oxidative phosphorylation and glycolysis [74]. Pitroda *et al* reported that knockdown of STAT1 with shRNA led to significant growth suppression of irradiated tumors and improved radiosensitization in human squamous cell carcinoma cell line SCC61, accompanied by alterations in glycolysis pathway [75]. Knockdown of PKM2 (a key regulator of glycolysis) with shRNA enhanced the radiosensitivity of A549 NSCLC cells *in vitro* and s.c xenografts *in vivo* [76]. The role of glycolysis pathway in radioresistance is shown in Figure 3. Lactate dehydrogenase A (LDHA) is a main metabolic enzyme for lactate production which is a terminal product from glycolysis. It plays an essential role in the glycolysis. Our recent findings indicated that increased LDHA was found in CaP RR cells and knockdown of LDHA with siRNA or specific inhibitor FX-11 can increase radiosensitivity in CaP RR cells (unpublished data), suggesting LDHA is associated with CaP radioresistance. Thus, targeting glycolysis pathway is likely to have broad therapeutic applications for cancer radioresistance.

### VEGF pathway

Angiogenesis is involved in pathogenesis, progression and metastasis in a variety of solid tumors. VEGF signaling pathway that is stimulated by upstream activators including environmental cues, growth factors, oncogenes, cytokines and hormones is a critically important growth factor pathway which stimulates vasculogenesis and angiogenesis. Overexpression of VEGF contributes to the growth and metastasis of solid tumors and inhibition of VEGF pathway offers potential clinical treatment for patients with hematologic malignancies [77]. The transcriptional regulation of VEGF pathway is associated with radiation by up-regulation of HIF-1α and NF-κB in CaP [78, 79]. It was found that the expression of HIF-1α/VEGF-A was suppressed in improved radiosensitivity in endometrial cancer cell lines HEC-6 (TP53 wild-type) and HEC-1B (TP53 mutant) [80]. Tian *et al* showed that eukaryotic translation initiation factor 4F (eIF4F) complex played a pivotal role in regulation of radiosensitivity by controlling the expression of VEGF and basic fibroblast growth factor (bFGF), two well-known pro-angiogenic factors [81]. Up-regulation of VEGF expression was shown in radiation induced murine squamous cancer cells NR-S1 [82]. Blocking radiation-induced VEGF pathway in CaP was found to interfere with tumor growth [45]. Several similar pieces of evidence were also demonstrated in HNSCC. The release of VEGF protein is increased during RT in HNSCC, which could be important for therapy success [64, 83]. The role of VEGF pathway in radioresistance is shown in Figure 3. In our recent studies, we also found the activation of this pathway with increased expression of VEGF VG-1 and VEGF R2 in PC-3RR CaP cell line and PC-3RR CaP xenograft mouse model (Figure 4). Thus, radiation-induced changes in VEGF pathway may modulate microenvironment and influence responsiveness of tumors to RT.

### Notch pathway

Notch pathway is a well-known CSC pathway [84]. This pathway is a highly conserved cell signaling system present in a wide range of human tumors such as leukemia [85], breast cancer [86], and glioma [87] through regulating self-renewal and repressing differentiation [88, 89]. It was reported that Notch pathway was activated in endothelial cells by IR shifting up-regulation of Jag1 and Hey1 [90]. High Notch pathway activity showed correlation with poor prognosis and radioresistance in NSCLC patients [91]. The result that inhibition of Notch pathway with gamma-secretase inhibitors (GSIs) rendered the glioma stem cells more sensitive to radiation at clinically relevant doses, suggests a critical role of Notch signaling to regulate radioresistance of glioma stem cells [92]. According to Zhang *et al*, inhibiting Notch-1 could regulate radiation-induced epithelial-mesenchymal transition (EMT) in gastric cancer cells [93]. Inhibiting radiation-induced Notch-1 signaling was found to enhance the RT efficacy in NCI-H1299 and NCI-H460 NSCLC cell lines *in vitro* and NCI-H1299 s.c xenografts *in vivo* [94]. It was recently reported that the non-adherent anoikis-resistant stem cell-like CaP cell population after RT showed activation of the Notch pathway and increased expression of stem cell markers CD133, Oct-4, Sox2 and Nanog [95]. All results suggest that Notch pathway inhibition could be developed as an adjuvant approach to improve current radiation treatment for human cancer. The role of the Notch pathway in cancer radioresistance is shown in Figure 3.

### Wnt/β-catenin pathway

Wnt/β-catenin pathway is another CSC pathway [84]. This pathway was reported to be associated with cancer metastasis in breast, lung and prostate cancers [96-98]. This pathway was also found to be involved in CSC radioresistance [99]. The role of the Wnt/β-catenin pathway in radioresistance is demonstrated in Figure 3. Su *et al* showed that the up-regulation of the Wnt/β-catenin pathway is important for KYSE-150 RR esophageal cancer cells [100, 101]. The downregulation of miR-301a promoted radioresistance in RR esophageal cell line KYSE-150R through the upregulation of Wnt1, indicating that Wnt/β-catenin signal pathway plays an important role in radioresistance [100]. Bastos *et al* observed that the irradiated colorectal cancer cells Caco-2, HT-29 and HCT-116 showed increased Wnt/β-catenin-dependent TCF/LEF activity [102]. Inhibition of Wnt/β-catenin pathway could radiosensitize CaP cells through decreasing aldehyde dehydrogenase (ALDH) [103]. Targeting a downstream marker of Wnt/β-catenin pathway WISP1 could sensitize esophageal squamous cell carcinoma (ESCC) to irradiation [104]. Zhang *et al* showed that WISP1 facilitated its own expression in response to radiation, created a positive feedback loop and increased radioresistance [104]. Thus, the radiosensitivity of cancer cells can be enhanced by inhibiting the Wnt/β-catenin pathway.

In summary, several important signaling pathways are closely associated with CSCs and radioresistance. These pathways constitute important targets for new adjuvant treatment schedules with RT, with the goal of reducing the migratory and invasive potential of the remaining cancer cells after treatment. The roles of different signaling pathways in cancer radiation response are summarized in Table 2.

**Table 2:** Different signaling pathways in cancer radioresistance

Signaling pathway	Source	Cancer	Radiation dose	Validation	Reference
PI3K/Akt/mTOR pathway	PC-3, DU145, LNCaP cell lines	prostate cancer	6Gy	Western blot	[8]
	C57BL/6J female mice xenograft	colorectal cancer	2Gy	QRT-PCR, flow cytometry, IHC, immunofluorescence	[47]
	PC-3, DU145, LNCaP cell lines	prostate cancer	2Gy	Western blot, ELISA	[11]
	PC-3 cell line	prostate cancer	4Gy	Flow cytometry, Western blot	[48]
	A549, H1299 cell lines, A549 subcutaneously xenograft	lung cancer	25 or 50 Gy	IHC, Western blot, RT-PCR	[50]
	H1299, H226B, H226Br, H460, H182 cell lines, animal xenograft	lung cancer	3Gy	Western blot, RT-PCR, immunoprecipitation	[52]
	CNE-2, 5-8F, 6-10B cell lines	nasopharyngeal cancer	4Gy (cells); 2 Gy every other day for four treatments (xenograft)	IHC, TUNEL	[54]
	HCT 116, SW 620, HT 29 cell lines, HCT116 subcutaneously xenograft	colorectal cancer	5Gy/fraction, three times in one week	IHC, Western blot	[55]
ERK pathway	HCT116, HT29, RKO, HCT15 cell lines	colorectal cancer	6Gy	Western blot, qRT-PCR	[57]
	H460, H1299 cell lines	non-small-cell lung cancer	6Gy	Western blot, qRT-PCR	[57]
	U87MG, T98G cell lines	glioblastoma	6Gy	Western blot, qRT-PCR	[57]
	769P, ACHN cell lines	clear cell renal cell cancer	6Gy	Western blot, qRT-PCR	[57]
	MDA-MB-231, MCF7 wild type, MCF7/HER2, MCF7/C6 cell lines	breast cancer	10Gy	Western blot, immunoblotting	[58]
	KYSE-150 cell line	esophageal cancer	2Gy or 6Gy	Western blot, immunoblotting, immunofluorescence	[59]
	A375 cell line	melanoma	8Gy	Western blot, flow cytometry, ROS	[60]
	HeLa cell line	cervical cancer	8Gy	Western blot, flow cytometry, ROS	[60]
	T98G, U138MG cell lines	glioblastoma	4Gy	Immunoblotting, Western blot	[61]
	293T, HeLa, C33A cell lines	cervical cancer	6Gy	Immunoprecipitation, Western blot, RT-PCR	[62]
	HeLa, CASKI, SiHa cell lines	cervical cancer	4Gy	Western blot, flow cytometry	[63]
	HNSCCUM-02T cell line	head and neck squamous cell cancer	30Gy	ROS, SDS-PAGE, Western blot, ELISA, IHC	[64]
	T24 cell line	bladder cancer	1.6Gy	Western blot	[66]
Glycolysis pathway	HeLa cell line	cervical cancer	2Gy	2D-LC-MS/MS, Western blot	[72]
	HepG2 cell line	liver cancer	2Gy	Immunofluorescence, Western blot, ROS	[73]
	HeLa cell line	cervical cancer	2Gy	Immunofluorescence, Western blot, ROS	[73]
	OE33 cell line	oesophageal adenocarcinoma	2Gy	IHC, Western blot, qRT-PCR	[74]
	OECM1, KB, SAS cell lines	head and neck cancer	60Gy	Western blot, flow cytometry, ROS	[105]
	CAL-33; FaDu; HSC-4; SAS; UT-SCC-5; UT-SCC-8; UT-SCC-14; UT-SCC-15; UT-SCC-45; XF354 xenografts	head and neck squamous cell cancer	4Gy per day for two days	IHC, Western blot	[106]
	SCC61 cell line	head and neck squamous cell cancer	5 Gy per day for six consecutive days	Affymetrix arrays	[75]
	NSCLC cell lines including A549, H460, H1299, H292, and H520, A549 xenograft	lung cancer	4Gy (cells), 4Gy per day for 7 days (xenograft)	Western blot, IHC	[76]
	prostate cancer patients	prostate cancer	a total of 15 fractions or 3.5 Gy per fraction within 19 days	IHC	[107]
VEGF pathway	HEC-108, HEC-6, HEC-151, Ishikawa, HEC-59, HEC-50B, HEC-1B, HEC-116 cell lines	endometrial cancer	10Gy	qRT-PCR, Western blot	[80]
	NR-S1 xenograft	head and neck squamous cell cancer	30, 50 or 70Gy	IHC, qRT-PCR	[82]
	PC-3, C4-2B cell lines	prostate cancer	3Gy	Immunofluorescence, Western blot	[45]
	HNSCCUM-02T cell line	head and neck squamous cell cancer	30Gy	ROS, SDS-PAGE, Western blot, ELISA, IHC	[64]
Notch pathway	H460 xenografts	lung cancer	10Gy	QRT-PCR, Western blot, IHC	[91]
	T3359, T3691, T4105, T4302, and T4597 cell lines	glioma	1, 2, or 3Gy	RT-PCR, Western blot	[92]
	NCI-H1299 and NCI-H460 cell lines, NCI-H1299 xenograft	lung cancer	2Gy (cells), 10Gy (xenograft)	Flow cytometry, qRT-PCR, Western blot, IHC, Immunofluorescence, northern blotting	[94]
Wnt/β-catenin pathway	KYSE-150 cell line	esophageal cancer	12 times (1 Gy three times, 2 Gy three times and 4 Gy three times) twice a week to a total dose of 21 Gy for 1.5 months	RT-PCR, Western blot	[100]
	Caco-2 (HTB-37™), HT-29 (HTB-38™), HCT-116 (CCL-247™) cell lines	colorectal cancer	5Gy	Immunoblotting, Immunofluorescence, qRT-PCR	[102]
	PC-3,DU145, and LNCaP cell lines, ALDH^+^and ALDH^−^DU145 xenografts	prostate cancer	4Gy per fraction to a total dose of 56 Gy (cells), 4Gy(xenografts)	Flow cytometry, Western blot, Immunofluorescence	[103]
	KYSE-150 cell line, KYSE-150 xenograft	esophageal cancer	6Gy (cells), 12Gy in three fractions every four days (xenografts)	Immunofluorescence, ELISA, IHC, Western blot	[104]

Notes: IHC, immunohistochemistry; qRT-PCR, Quantitative real time-PCR; ROS, reactive oxygen species; RT-PCR, reverse transcription polymerase chain reaction; SDS-PAGE, sodium dodecyl sulfate polyacrylamide gel electrophoresis; 2D LC-MS/MS, two-dimensional liquid chromatography-tandem mass spectrometry.

### Proteomic technologies in cancer RR signaling pathway research

**Figure 5:**
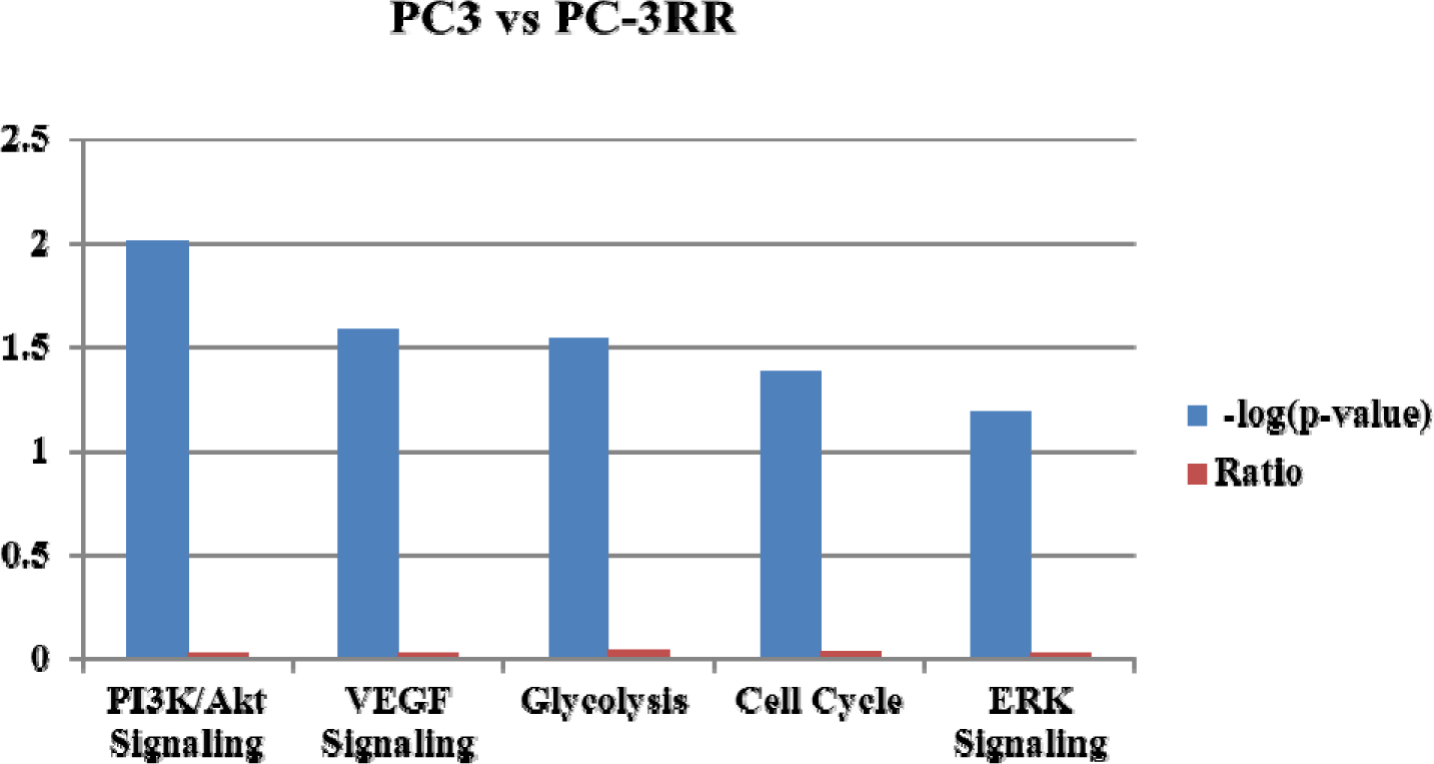
The top five potential pathways associated with prostate cancer radioresistance identified by label-free LC-MS/MS approach The *p* value and ratio of the identified top five potential pathways are displayed by Ingenuity pathway analysis between PC-3 and PC-3RR cells. The identified top five signaling pathways are PI3K/Akt, VEGF, glycolysis, cell cycle and ERK.

During the past few years, emerging proteomics technologies have been applied to study signaling pathways associated with cancer radioresistance. These proteomics techniques include two-dimensional difference gel electrophoresis (2D-DIGE), liquid chromatography-tandem mass spectrometry (LC-MS/MS) and multiple reaction monitoring (MRM). Using 2D-DIGE approache, Skvortsova *et al* compared the protein differences between three CaP RR cell lines (PC3-RR, DU145-RR and LNCaP-RR) and their parental cells to examine the involved signaling pathways and found CaP RR cells exhibited higher levels of androgen and epidermal growth factor (EGF) receptors and activation of their downstream pathways, including MAPK and PI3K/Akt and Jak-STAT [11]. In our recent study, total 309 signaling pathway proteins were identified to be significantly different between CaP RR (PC-3RR, DU145RR and LNCaPRR) and control (PC-3, DU145 and LNCaP) cells using the label-free LC-MS/MS method (unpublished data). Our results demonstrated that 5 signaling pathways including PI3K/Akt/mTOR, ERK, glycolysis, VEGF, as well as cell cycle were closely associated with CaP radioresistance (Figure 5). These technologies provide us with a broad vision and confident results to examine signaling pathway networks or new signaling pathway proteins in cancer radioresistance.

## CONCLUSIONS AND FUTURE DIRECTIONS

CSC and signaling pathway research continue to be an expanding field. The mechanisms of cancer radioresistance are still not fully uncovered. A better understanding of CSCs and associated signaling pathways that regulate radioresistance will hopefully open a new field of treatment strategies for cancer RT. The recent identity of CSCs has unlocked a new potential avenue for radioresistance research. Elucidating the role of CSCs and signaling pathways in the cancer cells' response to radiation will enhance our understanding of cancer recurrence after RT, and may direct research towards novel and specific radiosensitization agents that target CSCs or these pathway proteins.

Future clinical research designs should consider prospectively incorporating pre-treatment tumor biopsies during and after the RT course to track CSC phenotypes or signaling pathway proteins in a temporal manner. We expect that there will be increased understanding of the intrinsic and extrinsic factors that control the plasticity and maintenance of the CSC state. Due to complex and dynamic processes during fractionated RT, different RR signaling pathways may be better targeted at different stages of therapy to reduce the dependence of dose escalation. In addition, new inhibitors (radiosensitizers) should be developed to selectively target CSC and cancer-associated signaling pathway, and to reduce the toxicity to normal tissues. In the long term, any therapeutic strategy in RT will need to be taken into account of the biological features that control CSC behaviour and signaling pathway mechanisms to allow the implementation of personalized therapy.
